# Dense Hydrogen-Bonding Network Boosts Ionic Conductive Hydrogels with Extremely High Toughness, Rapid Self-Recovery, and Autonomous Adhesion for Human-Motion Detection

**DOI:** 10.34133/2021/9761625

**Published:** 2021-04-15

**Authors:** Bing Zhang, Xu Zhang, Kening Wan, Jixin Zhu, Jingsan Xu, Chao Zhang, Tianxi Liu

**Affiliations:** ^1^State Key Laboratory for Modification of Chemical Fibers and Polymer Materials, College of Materials Science and Engineering, Innovation Center for Textile Science and Technology, Donghua University, Shanghai 201620, China; ^2^School of Engineering and Materials Science, Queen Mary University of London, Mile End Road, London E1 4NS, UK; ^3^Shaanxi Institute of Flexible Electronics (SIFE), Northwestern Polytechnical University (NPU), 127 West Youyi Road, Xi'an 710072, China; ^4^School of Chemistry, Physics and Mechanical Engineering, Queensland University of Technology, Brisbane, QLD 4001, Australia; ^5^Key Laboratory of Synthetic and Biological Colloids, Ministry of Education, School of Chemical and Material Engineering, Jiangnan University, Wuxi 214122, China

## Abstract

The construction of ionic conductive hydrogels with high transparency, excellent mechanical robustness, high toughness, and rapid self-recovery is highly desired yet challenging. Herein, a hydrogen-bonding network densification strategy is presented for preparing a highly stretchable and transparent poly(ionic liquid) hydrogel (PAM-r-MVIC) from the perspective of random copolymerization of 1-methyl-3-(4-vinylbenzyl) imidazolium chloride and acrylamide in water. Ascribing to the formation of a dense hydrogen-bonding network, the resultant PAM-r-MVIC exhibited an intrinsically high stretchability (>1000%) and compressibility (90%), fast self-recovery with high toughness (2950 kJ m^−3^), and excellent fatigue resistance with no deviation for 100 cycles. Dissipative particle dynamics simulations revealed that the orientation of hydrogen bonds along the stretching direction boosted mechanical strength and toughness, which were further proved by the restriction of molecular chain movements ascribing to the formation of a dense hydrogen-bonding network from mean square displacement calculations. Combining with high ionic conductivity over a wide temperature range and autonomous adhesion on various surfaces with tailored adhesive strength, the PAM-r-MVIC can readily work as a highly stretchable and healable ionic conductor for a capacitive/resistive bimodal sensor with self-adhesion, high sensitivity, excellent linearity, and great durability. This study might provide a new path of designing and fabricating ionic conductive hydrogels with high mechanical elasticity, high toughness, and excellent fatigue resilience for skin-inspired ionic sensors in detecting complex human motions.

## 1. Introduction

A skin-inspired ionic sensor is widely concerned in the next generation of smart wearable electronics for the applications of artificial intelligence, human-machine interfaces, healthcare monitoring, and soft robotics [1–3]. An ionic sensor is capable of sensing external stimulations and transforming them into conductivity signals rapidly and in real time by imitating human skin [4]. The real-time response of an ionic sensor is realized through the directional migration of ions in an ionic conductor under deformation, which can realize the integrated functions of high elasticity and skin comparable modulus that are difficult to realize in a traditional electronic conductor. Due to the frequent movement of the human body and its rough and complex surface, ion sensors would inevitably be damaged and fall off during long-term wearing, which puts forward high requirements for tailored adhesive performances of ionic sensors [5, 6]. However, traditional adhesives are very difficult to meet the requirements of ionic sensors in long-term wearing or multiple-time adhering. Moreover, for their practical applications of human-machine interfaces, intelligent windows, and touchscreens, not only the ability to perceive external stimuli but also a high transmittance to achieve an output of visual information is required for an ionic sensor [7]. Therefore, the development of an ionic sensor with high transparency, high mechanical robustness, adaptive self-adhesion, and long cycling life is extremely demanded.

Poly(ionic liquid)s (PILs) are polymers formed through polymerization of ionic liquid (IL) monomers that feature repeated anionic or cationic groups. PIL hydrogels are capable of combining unique integrations of attractive mechanical characteristics of hydrogels and superior physicochemical properties of ILs [8–10]. PIL hydrogel is more thermally stable with strongly locked counter ions, and these features can help to overcome the leakage and poor environmental tolerance of conventional salt-impregnated polymer hydrogels. However, PIL hydrogel usually has poor mechanical ductility, leading to irreversible mechanical failures and poor cycling stability under large deformation. More importantly, PIL hydrogel is difficult to achieve high and tailored adhesion on various surfaces. Therefore, solving the problems mentioned above, i.e., achieving high mechanical elasticity, excellent fatigue resistance, and self-adhering performance, is necessary for the wide applications of PIL hydrogels for high-performance ionic sensors [11–13].

The human body mainly relies on ion channels inside the neurons to transmit information, and an electrolyte in organisms as an ionic conductor plays an essential role [14, 15]. This study attempts to construct a highly stretchable ionic conductive hydrogel to imitate the sensing functions of the skin. Herein, a highly stretchable and transparent poly(1-methyl-3-(4-vinylbenzyl) imidazolium chloride)-random-polyacrylamide copolymer hydrogel (PAM-r-MVIC) is fabricated by a hydrogen-bonding network densification strategy. Ascribing to the formation of a dense hydrogen-bonding network, the resultant PAM-r-MVIC exhibits excellent ductility with a large fracture strain (>1000%), along with high tensile strength of 0.47 MPa, high toughness of 2950 kJ m^−3^, and excellent fatigue resistance with no deviation for 100 cycles. Dissipative particle dynamics simulations further reveal that the mechanical properties are enhanced by the orientation of the dense hydrogen-bonding network along the stretching direction. Besides, mean square displacement calculations indicate that the high density of hydrogen bonds displays a large restriction of molecular chain movements, which also leads to dramatic enhancements in the mechanical strength. The positively charged 1-N atoms of imidazole rings among the PAM-r-MVIC are beneficial for uniformly locking counter ions, contributing to excellent ionic conductivity in a wide temperature range. Due to its high mechanical elasticity, high ionic conductivity, good transparency (close to 100% in visible light range), and excellent self-adhering properties, the PAM-r-MVIC can readily work in a resistive/capacitive bimodal sensor, showing high sensitivity, wide response range, and excellent stability in real-time monitoring of large-strain movements (i.e., finger and wrist bending) and small-strain movements (i.e., swallowing) of complex human motions. Therefore, this newly developed hydrogen-bonding network densification strategy for the design and construction of functional ionic conductive hydrogels provides new ideas for the development of ionic skin sensors with high mechanical elasticity, good transparency, self-adhering property, and excellent durability in a wide temperature range.

## 2. Results

**Figure 1:**
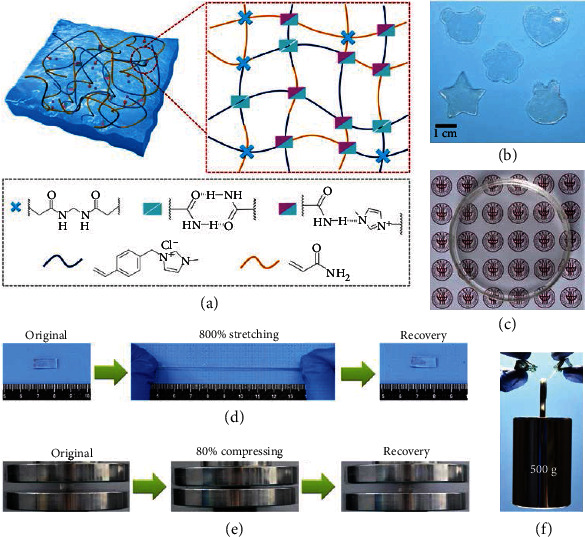
Design and properties of the PAM-r-MVIC. (a) Schematic illustration of PAM-r-MVIC with a dense hydrogen-bonding network. (b) Photograph of PAM-r-MVIC-2 fabricated into various shapes. (c) Photograph showing high transparency of PAM-r-MVIC-2. Photographs of PAM-r-MVIC-2 to withstand (d) large stretching and (e) compressing. (f) Photograph of PAM-r-MVIC-2 holding a weight of 500 g.

The design principle of constructing highly stretchable ionic conductive hydrogels by a hydrogen-bonding network densification strategy is demonstrated in Figure 1(a). Ionic conductive poly(1-methyl-3-(4-vinylbenzyl) imidazolium chloride)-random-polyacrylamide copolymer hydrogel (PAM-r-MVIC) was synthesized by in situ covalent crosslinking of 1-methyl-3-(4-vinylbenzyl) imidazolium chloride (MVIC) and acrylamide (AM) as comonomers. Hydrogen bonds between the amino groups among the AM structure and the 3-N atoms among the MVIC structure were achieved among the PAM-r-MVIC. Meanwhile, hydrogen bonds between the amino and carbonyl groups among the AM structure were simultaneously achieved within the PAM-r-MVIC. As a result, these two types of coexisted hydrogen bonds contributed to the formation of a dense hydrogen-bonding network among the PAM-r-MVIC. The as-fabricated hydrogel samples are named PAM-r-MVIC-1, PAM-r-MVIC-2, and PAM-r-MVIC-3, with the mole ratio of AM/MVIC varying from 2 : 1 and 1 : 1 to 1 : 2, respectively. Synthetic details of the synthesis route of MVIC could be found in Materials and Methods and Figure S1. MALDI-TOF results of as-synthesized MVIC (Figure S2) indicated the presence of a significant peak at 198.9 m/z, consistent with the molecular weight of MVIC. ^1^H NMR spectra of MVIC in D_2_O (Figure S3) displayed highly resolved signals corresponding to the skeleton protons, proving the successful synthesis of MVIC monomers [16].

The resultant PAM-r-MVIC can be easily molded into a variety of shapes of a bear, a heart, a hexaphyllum, a star, and a rabbit head, as demonstrated in Figure 1(b). Meanwhile, the as-obtained PAM-r-MVIC also exhibited high transparency, and the university logos behind a PAM-r-MVIC-2 film could be observed clearly (Figure 1(c)). Under a visible light with a wavelength range of 400~750 nm, the transmittance of the PAM-r-MVIC-2 film was higher than 90% (Figure S4). This high transparency of ionic conductors is conducive to monitoring the matrix changes in real time [17]. The PAM-r-MVIC-2 was further characterized by tensile and compression measurements (Figures 1(d) and 1(e)). The PAM-r-MVIC-2 is capable of being stretched to a strain of 800% and compressed to 80%, respectively, both with high toughness. The PAM-r-MVIC-2 with a dimension of 10 × 5 × 1 mm^3^ could pull up a weight of 500 g without any damages (Figure 1(f)). Notably, the shape of PAM-r-MVIC-2 could be recovered immediately after removing external forces, indicating its outstanding shape-recovery performance.

**Figure 2:**
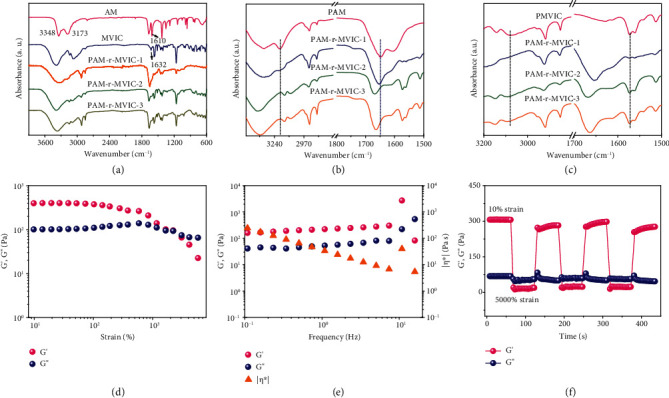
Interactions and rheological behaviors of the PAM-r-MVIC. (a) FTIR spectra of PAM-r-MVIC and monomers. (b) FTIR spectra of PAM-r-MVIC and PAM indicating peaks of imidazole rings. (c) FTIR spectra of PAM-r-MVIC and PMVIC indicating peaks of amino and carboxyl groups. (d) Strain sweep, (e) oscillatory shear, and (f) continuous step strain tests for PAM-r-MVIC-2.

The chemical structures of the hydrogels and the formation of dense hydrogen bonds in the PAM-r-MVIC were analyzed by Fourier Transform Infrared (FTIR) spectroscopy. The C=C stretching vibration of carboxyl groups at 1610 cm^−1^ from AM and the absorption peak located at 1632 cm^−1^ from MVIC disappeared (Figure 2(a)), indicating that the polymerization occurred for AM and MVIC monomers. The C=O stretching vibrations of carboxyl groups from the PAM, PAM-r-MVIC-1, PAM-r-MVIC-2, and PAM-r-MVIC-3 were observed at 1649, 1652, 1667, and 1663 cm^−1^, respectively, and the N-H stretching vibration peaks were observed at 3188, 3173, 3147, and 3144 cm^−1^, respectively (Figure 2(b)). The displacements of C=O and N-H stretching vibration peaks indicated the formation of hydrogen bonds among the PAM-r-MVIC. The absorption peak at 1571 cm^−1^ ascribed to the skeleton vibration of imidazole rings of the neat MVIC shifted to 1574 cm^−1^ of the PAM-r-MVIC-3 (Figure 2(c)). These chemical shifts demonstrated the formation of hydrogen bonds between the 3-N and N-H among the PAM-r-MVIC. Swelling tests were conducted to calculate the equilibrium swelling ratio for evaluating the strength of the hydrogen-bonding network among the PAM-r-MVIC. The PAM-r-MVIC-1 displayed the highest equilibrium swelling ratios (~220) among the PAM-r-MVIC samples (Figure S5). With the increase of the MVIC content among the PAM-r-MVIC framework, the equilibrium swelling ratio decreased gradually, indicating that the introduction of MVIC groups significantly enhanced the hydrogen-bonding density as well as the intermolecular interactions among the PAM-r-MVIC [18, 19].

Rheological measurements were performed to estimate the viscoelastic behaviors of the PAM-r-MVIC. Amplitude sweep tests were performed to estimate the viscoelasticity of the PAM-r-MVIC-2 sample, with the curves of storage modulus (*G*′) and loss modulus (*G*^″^) as a function of strain. For the strains ranging from 10% to 1000% in the linear viscoelastic region, both *G*′ and *G*^″^ remained constant with the value of *G*′ greater than *G*^″^ (Figure 2(d)). This corresponded to a typical solid-like behavior of viscoelastic hydrogels and revealed a stable network that remained unbroken even under extremely large deformations [20–22]. With the strains increasing, *G*′ decreased while *G*^″^ was raised. The *G*′ and *G*^″^ curves intersected when the strain reached 1650%, where the solid-liquid transition point indicating the damage of the network started. The oscillation frequency sweep test at 0.1% strain depicted that *G*′ was higher than *G*^″^ in the entire frequency range (Figure 2(e)), demonstrating that an elastic dominant network existed in the PAM-r-MVIC-2. A dynamic rheology experiment was designed to study the self-healing behavior of the PAM-r-MVIC-2. *G*′ was higher than *G*^″^ at 10% strain (Figure 2(f)), revealing that the PAM-r-MVIC-2 remained in a hydrogel state, while *G*′ was lower than *G*^″^ when the strain increased to 5000%, demonstrating that the hydrogel state was being destroyed. Notably, when the shear strain returned to 10%, *G*′ and *G*^″^ recovered immediately and were close to the initial value, indicating a rapid self-healing ability of the PAM-r-MVIC-2.

**Figure 3:**
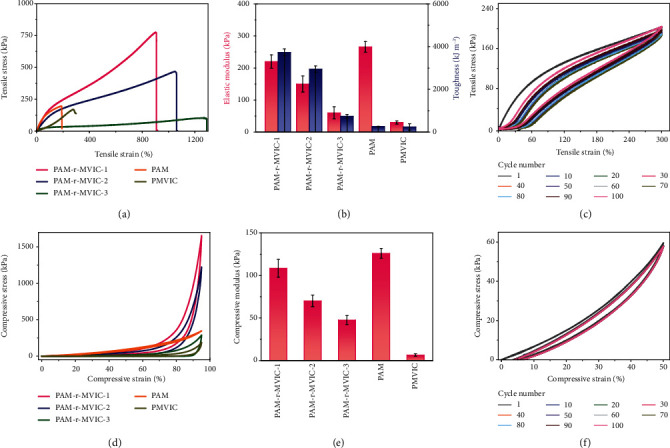
Mechanical properties of the PAM-r-MVIC. (a) Tensile stress-strain curves; (b) elastic modulus and toughness of PAM-r-MVIC, PAM, and PMVIC. (c) Successive tensile loading-unloading curves of PAM-r-MVIC-2. (d) Compressive stress-strain curves; (e) compressive modulus of PAM-r-MVIC, PAM, and PMVIC. (f) Successive compressive loading-unloading curves of PAM-r-MVIC-2.

Mechanical properties of PAM-r-MVIC were quantitatively analyzed to explore the effect of the intermolecular hydrogen bonds on their mechanical performance. Compared with PAM and PMVIC, the PAM-r-MVIC represented typical tensile stress-strain curves of elastomer and showed an outstanding mechanical performance including good elasticity, restorability, and fatigue resistance. Young's modulus of PAM and PMVIC was 266 and 30 kPa (Figure 3(a)), corresponding to the toughness of 256 and 240 kJ m^−3^, respectively. As the mole ratio of MVIC/AM increased from 0.5 to 2, the elastic modulus changed from 60 to 220 kPa, and the toughness was raised from 737 to 3731 kJ m^−3^ (Figure 3(b)). The enhancement of mechanical properties is attributed to the formation of a dense hydrogen-bonding network [23–26]. Hydrogels are easy to be broken when their moduli are too high, while hard to be stretched if they have too low toughness. Among the PAM-r-MVIC, the PAM-r-MVIC-2 showed the greatest balance among the modulus, toughness, and elongation at break. The 100 consecutive loading/unloading cycles under a 300% strain were carried out to estimate the fatigue resistance of PAM-r-MVIC-2 (Figure 3(c)). Meanwhile, the corresponding dissipation energy and dissipation coefficients were calculated (Figures S6a and S6b). The energy dissipation and recovery rate slightly decreased between the first and second laps but remained constant from the third lap. The stress-strain curves were also nearly overlapped from the 2^nd^ cycle to the 100^th^ cycle. This typical behavior of rubber elastomer was because the damaged network could be reconstructed in time during the tensile cycles [27, 28]. The first loading/unloading curve and after being stretched to 300% with the various resting times between two cycles were compared to characterize the self-recovery property (Figures S7a and S7b). Compared with the original sample, the tensile property of PAM-r-MVIC-2 increased with the increased resting time. After resting for 30 min, the dissipated energy and dissipation coefficient of the stretched PAM-r-MVIC-2 recovered to 107.5 kJ m^−3^ and 28.6%, approaching its original values (115.5 kJ m^−3^ and 26.6%). The excellent self-recovery ability manifested that the fractured networks could be rapidly repaired via reversible hydrogen-bonding networks [29, 30].

Compressive mechanical properties of PAM-r-MVIC hydrogels were also characterized to further evaluate their elasticity and self-recoverability. The PAM sample had a high compression modulus but poor toughness, while the PMVIC showed a low compression modulus, low toughness, and poor resilience (Figure 3(d)). Compared with PAM and PMVIC, the elastic modulus and toughness of the PAM-r-MVIC samples were significantly improved, indicating that the dense hydrogen bond network largely enhanced the mechanical properties of hydrogels. The PAM-r-MVIC-2 could withstand a 95% compression and recovered to its original state after relaxation. The compression moduli of PAM-r-MVIC-1, PAM-r-MVIC-2, and PAM-r-MVIC-3 were 108, 70, and 47 kPa, respectively (Figure 3(e)). The higher modulus normally led to more brittleness or lower toughness and thus worse resilience. With the increased content of MVIC, the compressive strength of PAM-r-MVIC gradually decreased, while their flexibility increased. The highly deformation-tolerant performance and excellent mechanical performance were ascribed to energy dissipation of the rapidly destroyed and rebuilt hydrogen bond networks [31]. Figure 3(f) demonstrates the continuous 100 loading-unloading curves of the PAM-r-MVIC-2 upon a 50% compressive strain. The hysteresis loops and loss factors of the PAM-r-MVIC-2 decreased slightly during the second compressive cycle. The dissipated energy and dissipation coefficient of the PAM-r-MVIC-2 at the 1^st^ and 2^nd^ cycles decreased from 24 to 18 kJ m^−3^ and from 0.2 to 0.17, respectively. This change indicated that the internal network of PAM-r-MVIC-2 broke down within the second time pressure and failed to recover in time. After resting for 0.5 min, it returned to the original state (Figure S7c), with the energy loss and loss factor consistent with the original state (Figure S7d). After the third cycle, the hysteresis loop and the loss factor of PAM-r-MVIC-2 almost remained. The dissipated energy and dissipation coefficient of the 3^rd^ and 4^th^ cycles remained around 17 kJ m^−3^ and 0.16, respectively, indicating a good fatigue resistance and highly deformation-tolerant and fast self-recovery performance [32–34]. The ionic conductive hydrogels with comprehensive mechanical properties demonstrated extraordinary stretchability and excellent toughness under different strains, thus showing a broad application prospect in flexible ionic devices.

**Figure 4:**
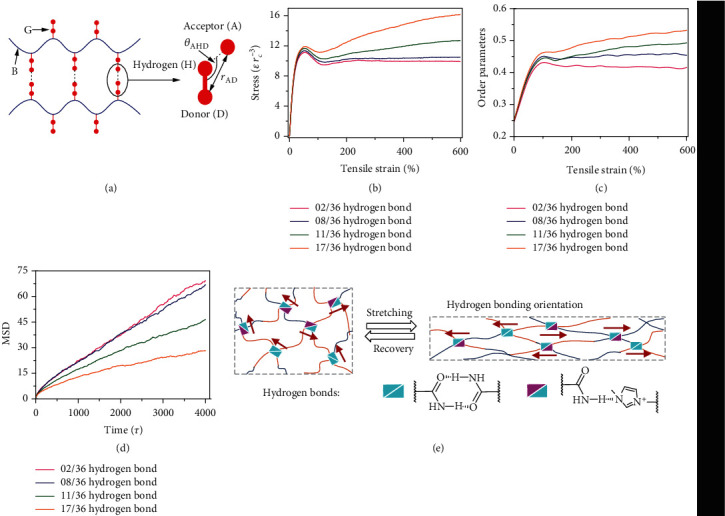
Simulation and calculation results of high stretchability of PAM-r-MVIC with a dense hydrogen-bonding network. (a) DPD model of PAM-r-MVIC with 5 graft arms (*n* = 5, denoted by 05/NB hydrogen-bonding system). (b) Stress-strain curves and (c) order parameters of molecular chain for PAM-r-MVIC with an increase of hydrogen-bonding contents under stretching. (d) Calculated MSD as a function of time for various PAM-r-MVIC (*n* = 2, 8, 11, and 17). (e) Schematic illustration of the orientation of hydrogen bonds and molecular chains during the stretching and recovery process.

To further describe the improved mechanical properties of the PAM-r-MVIC, dissipative particle dynamics (DPD) simulations were used to understand the mechanical strength evolutions (or stress-strain relationship) during the stretching.  Figure 4(a) shows the DPD model, among which each PAM-r-MVIC molecular chain consisted of a flexible backbone (B) grafted by a few (*n*) flexible grafts (G) symmetrically and uniformly (Table S1). G interacted with each other via hydrogen bonds. The enlarged view showed the hydrogen-bonding interaction described by the 3-body acceptor-hydrogen-donor (AHD) potential. *θ*_AHD_ was the angle among the donor, hydrogen, and acceptor beads, and *r*_AD_ was the radial distance between the donor and acceptor beads. The detail of the DPD simulation was demonstrated in supplemental materials. According to the DPD simulations, the tensile strength was enhanced when the content of hydrogen bonds increased under the tensile strain exceeding 50% (Figure 4(b)), which was consistent with the actual mechanical testing. To get insight into the possible mechanism of the improved mechanical properties behind the increase in hydrogen-bonding contents, we further calculated the orientation degree (order parameters) and the mean square displacement (MSD) of the molecular chains based on the DPD simulations during the stretching in Figures 4(c) and 4(d), respectively. MSD is a physical parameter to measure the deviation of the position of a particle relative to its reference position as it moves over time, which could directly reflect the capacity of the movement of the molecular chains. It can be observed from Figure 4(c) and Figure S8 that the orientation degree (order parameters) showed an increase with the increasing density of hydrogen bonds, resulting in improved tensile strength. The MSD of PAM-r-MVIC increased linearly with time in the long-time-regime (Figure 4(d)), suggesting that the PAM-r-MVIC exhibited a diffusive behavior. Moreover, the diffusion coefficient *D*_*c*_ (the slope of MSD at long-time-regime) reduced when the density of hydrogen bonds increased, indicating that the high-density hydrogen bonds restricted the movement of chain segments and also led to the high tensile strength.  Figure 4(e) macroscopically describes the original state of the hydrogels in its disordered state, while the molecular chains gradually became oriented during the process of stretching and vice versa during the recovery process.

**Figure 5:**
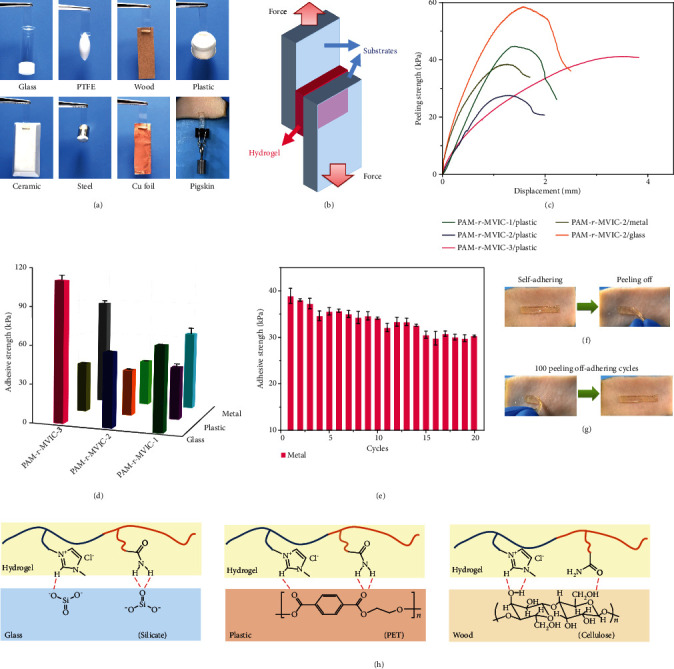
Self-adhesion performance of the PAM-r-MVIC. (a) Photographs of PAM-r-MVIC-2 self-adhered to various substrates. (b) Schematic illustration of lap shear tests. (c) Lap shear adhesion curves of PAM-r-MVIC on various substrates. (d) Adhesive mechanical strength of PAM-r-MVIC on various substrates. (e) Adhesive mechanical strengths of PAM-r-MVIC-2 to metals for various peeling off-adhering cycles. (f) Photographs showing the self-adhering and peeling of PAM-r-MVIC-2 on pigskin. (g) Photographs showing the peeling off-adhering processes for 100 cycles on pigskin. (h) Schematic illustration of the self-adhesion mechanism of PAM-r-MVIC on various substrates.

The as-obtained PAM-r-MVIC had a strong self-adhesiveness to various substrates, including glass, polytetrafluoroethylene (PTFE), wood, rubber, ceramics, steel, copper, and pigskin (Figure 5(a)). To quantitatively characterize the adhesive strength, a lap shear test was adopted to the hydrogel sample of PAM-r-MVIC-2, sandwiched between a pair of substrates, as shown in Figure 5(b). Figure 5(c) shows the adhesion strength-displacement curves on glass, plastic (polyethylene terephthalate (PET)), and metal (alloy). The peeling strength was taken as the interface adhesion strength for the interface failure, and the values of PAM-r-MVIC-1, PAM-r-MVIC-2, and PAM-r-MVIC-3 on the PET were 44.7, 27.3, and 41.0 kPa at 1.4, 1.2, and 3.5 mm, respectively. The peeling strength of PAM-r-MVIC-2 on metal was 38.4 kPa at 1.2 mm and on glass was 58.5 kPa at 1.58 mm. Compared with the various samples and substrates, the PAM-r-MVIC-3 adhering to the glass showed the highest adhesion strength (Figure 5(d)). After 20 repeated adhering and peeling cycles on metals, the PAM-r-MVIC-2 kept a good adhesion with the adhesive strength only decreasing from 40 to 30 kPa (Figure 5(e)). Excessive adhesion leads to substrate damage, or the lack of adhesive force leads to easily falling off, which are major obstacles to the applications of self-adhesive hydrogels in ionic skins [35, 36]. For the sake of this, the PAM-r-MVIC-2 was examined by adherence to pigskin. It was easy to be peeled without any residue or irritation (Figure 5(f)). After self-adhering and peeling off 100 times, the PAM-r-MVIC-2 still adhered well to the pigskin without damage (Figure 5(g)), indicating a reliable and repeatable adhesion for ionic skin applications.

The high and durable self-adhesion of the PAM-r-MVIC is attributed to the interaction of functional groups from the surface of hydrogels with substrates (Figure 5(h)). Carbonyl and amino groups could form covalent and noncovalent bonding on different surfaces. Between the PAM-r-MVIC and charged surfaces, dipole-dipole and ion-dipole interactions also existed. Taking the glass as an example, the surface of the glass was mainly composed of a double bond of silicon-oxygen and oxygen anion. Oxygen anion formed the ionic bond with imidazole cation, and dense hydrogen bonds were formed between the double bond of an amino group and silicon-oxygen, which dramatically enhanced the cohesion of the PAM-r-MVIC. The ionic bond played a leading role when the ion content was high in the PAM-r-MVIC-3. On the contrary, the number of hydrogen bonding was larger than that of ionic bonds in PAM-r-MVIC-1. With the increase of ion content, the hydrogen-bonding interaction gradually weakened and the dipole-dipole interaction became stronger as the bond energy of ionic bond was greater than hydrogen bond, resulting in the adhesive strength of glass increasing at first then decreasing. A similar mechanism also existed for the adhesion of PAM-r-MVIC to other substrates such as plastic and wood. The PET plastic mainly contained carbon-based and oxygen, which formed hydrogen bonding with hydrogen in the PIL hydrogel and formed an ion-dipole interaction with imidazole ions. As for wood, the hydroxyl groups on cellulose formed hydrogen bonding with nitrogen and carbonyl groups, and oxygen on cellulose forms ion-dipole interaction with imidazole ions. The triple interaction between cellulose and PAM-r-MVIC led to the adhesion strength which was better than those of glass and plastic. Therefore, the substrate surface has enough surface energy to achieve excellent bonding performance at the bonding interface, where the PAM-r-MVIC had a high adhesion strength and repeatable adhesion.

**Figure 6:**
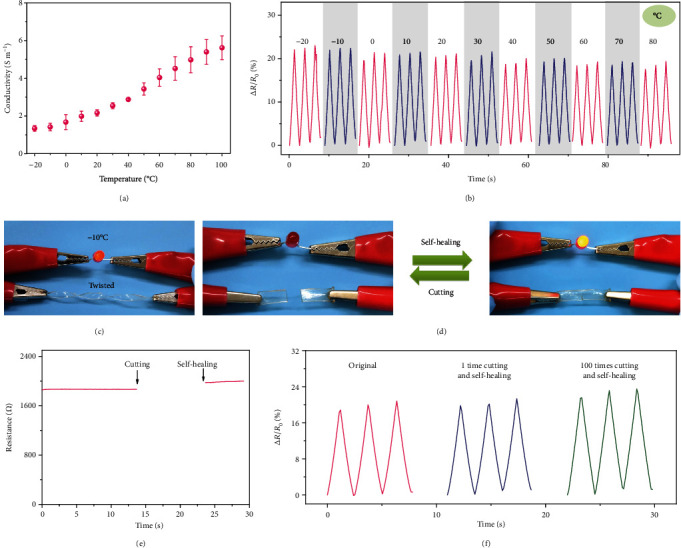
Extreme temperature tolerance and self-healing performance of the PAM-r-MVIC-2. (a) Temperature dependence of ionic conductivity from -20 to 100°C. (b) Relative resistance variation at various temperatures. (c) Photograph showing high flexibility at a low temperature. (d) Photograph showing the cutting and self-healing processes of PAM-r-MVIC-2 when connected to a circuit for lightening up a bump. (e) Resistance before and after cutting and self-healing. (f) Relative resistance variation after cutting and self-healing processes for various times.

The ionic conductivity of PAM-r-MVIC-2 was investigated over a wide temperature range, and its ionic conductivity increased from 1.3 to 5.6 S m^−1^ from -20 to 100°C (Figure 6(a)). Ionic conductivities were enhanced as a result of fast ion movements with an increasing temperature [37–39]. Figure S9a depicted that the resistance values gradually decreased as temperature increased from 25 to 70°C, which was consistent with the results of the varied ionic conductivity as the temperature increased. Besides, the PAM-r-MVIC-2 exhibited a promising ionic conductivity even at an extremely low temperature (Figure S9b), attributed to a reduced freezing point of hydrogels by the introduction of PIL backbones [40–43]. The sensing performance over a wide temperature range (-20~80°C) was illustrated (Figure 6(b)). The output signals were stable but gradually decreased with the raised temperature, ascribing to an increase of ionic conductivity. The PAM-r-MVIC-2 was not frozen at -10°C and able to conduct to lighten up a light-emitting diode (LED) bulb (Figure 6(c)). After being cut into two pieces, the PAM-r-MVIC-2 self-healed immediately and lit up the LED bulb again (Figure 6(d)). The resistance of the PAM-r-MVIC-2 film was slightly increased from 1870 to 1970 *Ω* after being cut and self-healed (Figure 6(e)), indicating that the PAM-r-MVIC-2 not only repaired its mechanical damage but also restored the ionic conductivity. The sensing performance of the PAM-r-MVIC during cutting-healing cycles was furthered measured (Figure 6(f)). Δ*R*/*R*_0_ (%) after one-time healing was nearly consistent with the original sample. After 100 times of cutting and self-healing, the healed sample could still restore over 95% sensing performance compared to the original sample. This high-efficiency and reliable sensing capability of the PAM-r-MVIC-2 offered great potentials in the application of wearable sensors.

**Figure 7:**
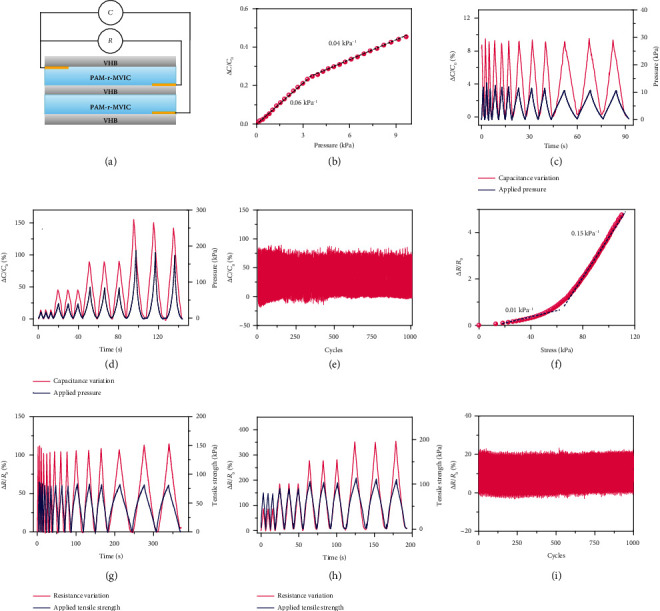
Sensing performance of the PAM-r-MVIC-2 in bimodal capacitive/resistive ionic sensors. (a) Schematic illustration for the design of bimodal sensors. (b) Sensitivities in a capacitance mode. Relative capacitance variation under pressures with various (c) frequencies and (d) forces. (e) Cycling stability of relative capacitive changes. (f) Sensitivities in a resistance mode. Relative resistance variation under pressures with various (g) frequencies and (h) forces. (i) Cycling stability of relative resistance changes.

Ascribing to the excellent mechanical elasticity and high ionic conductivity, the PAM-r-MVIC is an ideal candidate for wearable ionic skin sensors compared with ionic conductive gels in literature (Table S2). An ionic sensor in an integrated capacitance/resistance bimodal type was assembled using the PAM-r-MVIC-2 as a stretchable ionic conductor (Figure 7(a)). Benefitted from its self-adhesive properties, the PAM-r-MVIC could adhere tightly to the polyethylene dielectric layer without any gap, further improving the measurement accuracy. Sensitivity was defined from the relative resistance change (*R*‐*R*_0_/*R*_0_) and capacitance change (*C*‐*C*_0_/*C*_0_) versus stress and obtained from the slope of the curve [44–46]. *R*_0_ and *C*_0_ are the original resistance and capacitive, and *R* and *C* are the real-time resistance under stress, respectively. The capacitive sensitivity of the PAM-r-MVIC-2 ionic sensor was 0.06 kPa^−1^ when the stress was less than 3 kPa (Figure 7(b)). The sensitivity reduced to 0.04 kPa^−1^ under stress in a range of 3-9 kPa. Figure 7(c) demonstrates that the output capacitance signal and the input stress of PAM-r-MVIC-2 matched well and both waveform peaks synchronized, indicating a negligible signal hysteresis. When the pressure was applied to 10 kPa at different frequencies (0.03-0.5 Hz), Δ*C*/*C*_0_ (%) was kept consistent with its mechanical behavior and no electromechanical hysteresis was observed. Figure 7(d) shows capacitance response profiles under various pressures. The capacitances changed with the increased pressures, attributing to the ionic conductive path being significantly improved with increased pressure. As shown in Figure 7(e), 1000 loading/unloading cycles at a pressure of 0-40 kPa were applied to the PAM-r-MVIC-2 sensors, and Δ*C*/*C*_0_ (%) changed between 0 and 80% without any obvious signal deviation, indicating the excellent stability and repeatability.

The resistance-type sensing properties were also evaluated. The resistive sensitivity of the sensor was 0.01 kPa^−1^ and 0.15 kPa^−1^ with the applied tensile strength within the ranges of 0-50 kPa^−1^ and 50-110 kPa^−1^ (Figure 7(f)), respectively. The change of Δ*R*/*R*_0_ from 0 to 105% kept a good linear relationship within the stress range of 0-60 kPa (Figure 7(g)). The ionic sensors were able to respond to the continuous pressure with different loading speeds (0.03~0.5 Hz) and produced a good match between the pressure and the relative resistance change. The real-time responses were tested under cyclic loading/unloading pressures of 0-80, 0-90, 0-100, and 0-110 kPa (Figure 7(h)), and the corresponding relative resistance increased linearly with the change of 0-85%, 0-185%, 0-280%, and 0-350%, respectively, showing stable, continuous, and highly reproducible signals at various pressures. Subjecting the ionic sensor to 1000 compression/release cycles at a pressure of 0-10 kPa, the output signal was consistent with the applied pressure with ultrahigh stability (Figure 7(i)), attributing to its good mechanical durability and reproducibility. These results indicate that the assembled bimodal ionic sensor possesses the potential for dynamic consecutive pressure detection [47, 48].

**Figure 8:**
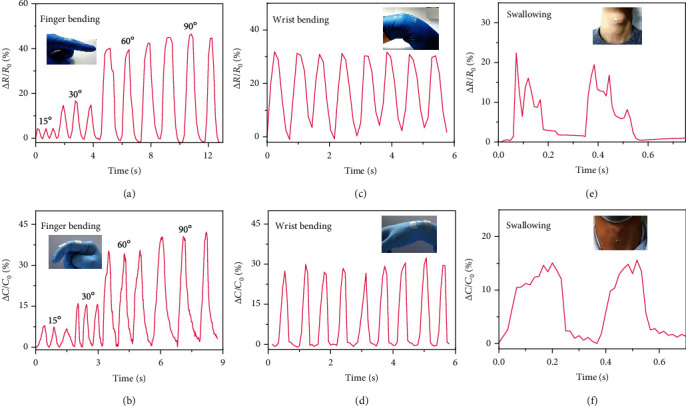
Wearable PAM-r-MVIC-2 bimodal sensors monitoring complex human motions. Real-time resistance and capacitance signals recorded in detecting (a, b) finger bending, (c, d) wrist bending, and (e, f) swallowing. Inset photographs were captured during corresponding measurements.

The potential application of PIL hydrogels for wearable ionic sensors was investigated considering that the PAM-r-MVIC integrated many desirable properties. The PAM-r-MVIC bimodal sensor was worn onto different parts of the human body, and the relative changes in resistance and capacitance during human activities were recorded. The various movements were accurately monitored under different pressure conditions. When the index finger stepwise bent to 15°, 30°, 60°, and 90°, Δ*R*/*R*_0_ increased to 4%, 14%, 40%, and 45% (Figure 8(a)), and Δ*C*/*C*_0_ increased to 7%, 15%, 35%, and 40% (Figure 8(b)), respectively. When PAM-r-MVIC-2 was stuck on the wrist and swung regularly at an angle of 30°, Δ*R*/*R*_0_ (Figure 8(c)) and Δ*C*/*C*_0_ (Figure 8(d)) gradually increased to 31% and 28%, respectively. Apart from the ability to perceive large human body motions, the PAM-r-MVIC-2 stress sensors could also monitor subtle deformation. When adhering the encapsulated PAM-r-MVIC-2 to the throat of an adult, the signals could be reflected when swallowing occurred (Figures 8(e) and 8(f)). These repeatable signals powerfully demonstrated the reliability of PIL hydrogels as a flexible wearable sensor [49, 50].

## 3. Discussion

A novel PAM-r-MVIC hydrogel was prepared by a hydrogen-bonding network densification strategy, during which a chemically cross-linked random copolymer with an intermolecular dense hydrogen-bonding interaction was in situ formed. Benefitted from the imidazole groups in the hydrogel backbone and dense hydrogen-bonding network, the resultant hydrogels combined outstanding mechanical properties (e.g., high strength, stretchability, compressibility, toughness, fast self-recovery, and fatigue resistance), high transparency of nearly 100% in the visible light range, and excellent ionic conductivity. The DPD simulations and MSD calculations further proposed the mechanical enhancement mechanism for the PAM-r-MVIC hydrogel, manifesting that the orientation of hydrogen bonds during the stretching and the restriction of molecular chains by the dense hydrogen-bonding network synergistically led to dramatically improved mechanical strength and toughness. More importantly, the PAM-r-MVIC hydrogel is capable of being adhered to diverse surfaces, such as metal, wood, plastic, glass, and skin, and the tailored adhesive mechanical strength was highly maintained after repeated adhering and stripping cycles for 100 times. Besides, the PAM-r-MVIC hydrogel is capable of maintaining high ionic conductivity, excellent compressive sensitivity, and high durability at extremely cold temperatures, allowing them to be designed as a capacitive/resistive bimodal sensor for human-motion detections. The PAM-r-MVIC sensors could accurately monitor both large-range human movements and small stress changes, such as the motions of finger bending, wrist flexion, and swallowing. It was envisioned that the hydrogen-bonding network densification strategy provided a new path for the preparation of ionic conductive hydrogels with high mechanical elasticity, excellent fatigue resilience, high sensitivity, and outstanding durability in a wide temperature range for skin-inspired ionic sensors.

## 4. Materials and Methods

### 4.1. Synthesis of the MVIC

1-Methylimidazol (2.1 g, 25 mmol), 4-vinylbenzyl chloride (3.82 g, 25 mmol), and DI water (1 mL) were mixed and stirred at 25°C in nitrogen for 12 h. Yellow oily liquid was collected and washed with diethyl ether and ethyl acetate, respectively. The products were freeze-dried in a vacuum to obtain a transparent and viscous liquid, and the yield is 2.99 g (~60.0%). ^1^H NMR (400 MHz, D_2_O) *δ* 8.62 (s, 1H), 7.26 (s, 1H), 7.22 (s, 1H), 7.18 (d, *J* = 7.8 Hz, 5H), 6.47 (dd, *J* = 17.6, 11.0 Hz, 2H), 5.61 (d, *J* = 17.7 Hz, 2H), 5.12 (s, 4H), 5.11 (s, 1H), 3.67 (s, 6H). MALDI-TOF for C_13_H_15_N_2_ [M]^+^: *m*/*z* = 198, found: 199.

### 4.2. Preparation of PAM-r-MVIC Hydrogels

A mixture of AM (1.06 g, 15 mmol), MVIC (2.985 g, 15 mmol), *N,N*′-methylene bisacrylamide (MBA, 4.6 mg, 0.03 mmol), and ammonium persulphate (APS, 6.8 mg, 0.03 mmol) was dissolved into 5 mL of DI water under stirring. The mixed solution was bubbled with nitrogen for 30 min and then poured into a PTFE mold at 50°C in a vacuum for 6 h. The PAM-r-MVIC was obtained, and the PAM-r-MVIC-1, PAM-r-MVIC-2, and PAM-r-MVIC-3 represent the as-obtained hydrogel samples with an AM/MVIC mole ratio of 2 : 1, 1 : 1, and 1 : 2, respectively. For comparison, hydrogel samples of PAM and PMVIC were prepared following the described method for the preparation of PAM-r-MVIC, but without the addition of MVIC and PAM, respectively.

### 4.3. Order Parameter

The order parameter of the stretched molecular chain during the stretching process is characterized by the second-order Legendre polynomials <*P*_2_> and is given by [51]

(1) $$\begin{array}{c} \langle P_{2}\rangle =\frac{\left(3\langle \cos^{2}\theta \rangle -1\right)}{2}, \end{array}$$

where *θ* is the angle between bonds and the deformed direction.

### 4.4. Mean Square Displacement and Diffusive Coefficient

The temporal evolution of the mean square displacement (MSD) as a function of time for different PAM-r-MVIC systems (i.e., *n* = 2, 8, 11, and 17) was obtained from the dissipative particle dynamics (DPD) simulations at the equilibrium state. The diffusion coefficient *D*_*c*_ could be deduced from the linear fitting of data in the long-time-regime:

(2) $$\begin{array}{c} \text{MSD}=\langle {\left[R\left(t\right)-R\left(0\right)\right]}^{2}\rangle ,\\ D_{c}=\frac{1}{6}\underset{t\to \infty }{\lim}\frac{d}{dt}\text{MSD}, \end{array}$$

where **R**(*t*) and **R**(0) are the position of the center-of-mass of the DPD bead at time *t* and 0, respectively.

## Data Availability

The data is available from the authors.

## Supplementary Materials

Supplementary Materials Supplemental information includes materials, characterizations, dissipative particle dynamics, 9 figures, and 2 tables. Figure S1: synthesis route of the MVIC. Figure S2: MALDI-TOF mass spectrum of the MVIC. Figure S3: 1H NMR spectrum of MVIC in D_2_O. Figure S4: UV-vis spectra of the PAM-r-MVIC. Figure S5: equilibrium swelling ratios of the PAM-r-MVIC in water. Figure S6: successive loading-unloading tests of PAM-r-MVIC under tensile and compression. Dissipated energy and energy dissipation coefficients of PAM-r-MVIC-2 under tensile/compressive cycles. Stress-strain curves of PAM-r-MVIC-2 at various tensile/compressive strains. Figure S7: tensile recovery test of PAM-r-MVIC-2 with various resting times. Tensile dissipated energy and energy dissipation coefficients during the tensile recovery test with various resting times. Compressive recovery test of PAM-r-MVIC-2 with various resting times. Tensile dissipated energy and energy dissipation coefficients during the compressive recovery test with various resting times. Figure S8: order parameter of hydrogen bonds with various contents during stretching. Figure S9: temperature-dependent resistance changes of PAM-r-MVIC-2. Photograph showing the PAM-r-MVIC-2 lightening up an LED bulb at an extremely low temperature. Table S1: interaction parameters *a*_*ij*_ (in DPD units) used in the simulations. Table S2: summary of mechanical properties and sensing performances of PAM-r-MVIC and ionic conductive gels in literature.
