# Supplementary material for: Dense Hydrogen-Bonding Network Boosts Ionic Conductive Hydrogels with Extremely High Toughness, Rapid Self-Recovery, and Autonomous Adhesion for Human-Motion Detection
Source: Research (Wash D C). 2021 Apr 15;2021:9761625. doi: 10.34133/2021/9761625 (PMC8067885; doi:10.34133/2021/9761625)
Supplement: Supplementary Materials — Supplemental information includes materials, characterizations, dissipative particle dynamics, 9 figures, and 2 tables. Figure S1: synthesis route of the MVIC. Figure S2: MALDI-TOF mass spectrum of the MVIC. Figure S3: 1H NMR spectrum of MVIC in D2O. Figure S4: UV-vis spectra of the PAM-r-MVIC. Figure S5: equilibrium swelling ratios of the PAM-r-MVIC in water. Figure S6: successive loading-unloading tests of PAM-r-MVIC under tensile and compression. Dissipated energy and energy dissipation coefficients of PAM-r-MVIC-2 under tensile/compressive cycles. Stress-strain curves of PAM-r-MVIC-2 at various tensile/compressive strains. Figure S7: tensile recovery test of PAM-r-MVIC-2 with various resting times. Tensile dissipated energy and energy dissipation coefficients during the tensile recovery test with various resting times. Compressive recovery test of PAM-r-MVIC-2 with various resting times. Tensile dissipated energy and energy dissipation coefficients during the compressive recovery test with various resting times. Figure S8: order parameter of hydrogen bonds with various contents during stretching. Figure S9: temperature-dependent resistance changes of PAM-r-MVIC-2. Photograph showing the PAM-r-MVIC-2 lightening up an LED bulb at an extremely low temperature. Table S1: interaction parameters aij (in DPD units) used in the simulations. Table S2: summary of mechanical properties and sensing performances of PAM-r-MVIC and ionic conductive gels in literature. [file 9761625.f1.docx]

**Supplementary Materials**

**Title**

**Dense hydrogen-bonding network boosts ionic conductive hydrogels with extremely high toughness, rapid self-recovery and autonomous adhesion for human-motion detection**

**Authors**

Bing Zhang^1^, Xu Zhang^1^, Kening Wang^3^, Jixin Zhu^4^, Jingsan Xu^5^, Chao Zhang^1^*, and Tianxi Liu^1,2^*

**Affiliations**

^1^State Key Laboratory for Modification of Chemical Fibers and Polymer Materials, College of Materials Science and Engineering, Innovation Center for Textile Science and Technology, Donghua University, Shanghai 201620, P. R. China

^2^Key Laboratory of Synthetic and Biological Colloids, Ministry of Education, School of Chemical and Material Engineering, Jiangnan University, Wuxi 214122, P. R. China

^3^School of Engineering and Materials Science, Queen Mary University of London, Mile End Road, London, E1 4NS UK

^4^Shaanxi Institute of Flexible Electronics (SIFE), Northwestern Polytechnical University (NPU), 127 West Youyi Road, Xi'an 710072, P. R. China

^5^School of Chemistry, Physics and Mechanical Engineering, Queensland University of Technology, Brisbane, QLD 4001, Australia

Correspondence should be addressed to Tianxi Liu; txliu@fudan.edu.cn and Chao Zhang; czhang@dhu.edu.cn

**Outline**

**S-1. Materials**

**S-2. Characterizations**

**S-3. Dissipative particle dynamics (DPD)**

**S-4. Supplementary figures**

**S-1. Materials**

4-vinylbenzyl chloride (MVIC, 90%) was purchased from Sigma-Aldrich. Acrylamide (AM, CP, ≥ 98.0%) and ammonium persulphate (APS, AR, ≥ 98.0%) were purchased from Sinopharm Chemical Reagent Co., Ltd. *N,N'*-Methylenebisacrylamide (MBA, 97%) was purchased from J&K Scientific Ltd. Deionized (DI) water was used for all experiments. All the reagents were used without further purification except the recrystallization of APS using DI water.

**S-2. Characterizations**

Chemical structures of MVIC were determined by ^1^H NMR and MALDI-TOF mass spectrometry. ^1^H NMR spectrum was recorded on a Bruker AV 400 NMR spectrometer (400 MHz). FTIR spectra in a wavenumber range from 4000 to 600 cm^-1^ were measured with a Thermo Nicolet 6700 FT-IR Laboratory Spectrometer. Mechanical properties were tested using a universal testing machine (Suns Tech.). Tensile and compressing rates were set at 100 and 10 mm min^-1^, respectively. All hydrogel samples were molded into a cuboid of 10 × 5 × 1 mm^3^ and a cylinder of Φ10 × 10 mm^3^ for tensile and compression tests, respectively. Thin-layered silicone oil was coated on hydrogel surfaces to reduce water evaporation before mechanical tests. Elastic moduli from the tensile and compression tests were determined by the slope of the stress-strain curve in the strain range of 0.05~0.15 mm mm^-1^.

Dissipated energy in the fatigue tests was calculated by the following equation:

*Ui= ∫_loading_σdε - ∫_unloading_σdε*

where *σ* and *ε* represent stress and strain, respectively.

The transparency of hydrogel samples with a thickness of 1 mm was measured with a TU-1901 UV-vis spectrophotometer.

Equilibrium swelling ratios of hydrogel samples were measured as follows: The as-obtained hydrogel samples were swollen in DI water until reaching an equilibrium at room temperature and the water was replaced every 1 h. Before weighing, the excess surface water was wiped off. The equilibrium swelling ratio was calculated according to the following equation.

$$Equilibrium swelling ratio = \frac{{W-W}_{0}}{W_{0}}$$

where *W_0_* and *W* represent the weight of hydrogels before and after swelling in water, respectively.

Rheological performance was measured using a rheometer (Anton paar MVR 302, Austria). Strain sweeping measurements were conducted at a strain from 0.1% to 5000% at a constant angular frequency of 5 rad s^-1^, and the angular frequency sweeping tests were performed from 0.1 to 100 rad s^-1^ at a constant strain of 10%.

Ionic conductivity of hydrogel samples was characterized by a conventional four-point method.

Adhesion performance of hydrogel samples was examined by lap-shear tests. The glass, plastic and metal substrates without contaminants were fabricated into splines in the sizes of 40 × 25 mm^2^. Hydrogel samples (25 × 25 × 1 mm^3^) were sandwiched between two substrates with an area of 25 × 25 mm^2^. A pressure of 100 g was applied for 5 min to form a tight contact on the joint area before testing. The lap-shear tests were performed with a rate of 10 mm min^-1^ until the joint was split. The waiting time for each attachment was set at 3 min in the repeated adhering and peeling cycles.

Capacitive/resistive bimodal sensor was assembled using two as-obtained hydrogel samples (10 × 5 × 1 mm^3^) and a 3M VHB 4905 film (thickness: 0.5 mm) as electrodes and dielectric layer, respectively. The capacitive and resistance were recorded by a Keithley 2400 digital source meter and LCR meter (TH 2832), respectively. Relative changes in capacitance and resistance were defined as the following equations.

$$\frac{\Delta C}{C_{0}}= \frac{{C-C}_{0}}{C_{0}}\times100$$

$$\frac{\Delta R}{R_{0}}= \frac{{R-R}_{0}}{R_{0}} \times100$$

Sensitivity was calculated from relative resistance changes (*ΔR/R_0_*) and relative capacitance changes (*ΔC/C_0_*) versus stress, respectively.

Capacitance of the bimodal sensor was calculated by the following equation.

$$C=\frac{\varepsilon A}{4\pi kd}$$

where *C* is the capacitance, *ε* and *d* are the dielectric constant and thickness of the dielectric layer, *k* is the electrostatic constant, and *A* is the effective area of hydrogel samples.

**S-3. Dissipative particle dynamics (DPD)**

In the DPD, the polymer molecules are modeled by bead-spring chains. All the beads possessing a mass of *m* represent a cluster of atoms and interact pairwise via bead-bead interactions. The force $\mathbf{f}_{i}$ acting on the *i*th bead includes the conservative force $\mathbf{F}_{ij}^{C}$, the dissipative force $\mathbf{F}_{ij}^{D}$, and the random force $\mathbf{F}_{ij}^{R}$, which is given by

$$\mathbf{f}_{i}\boldsymbol{=}\sum_{j\neq i} \left( \mathbf{F}_{ij}^{C}+\mathbf{F}_{ij}^{D}+\mathbf{F}_{ij}^{R} \right)$$

The conservative force is a soft repulsion taking the form of

$$\mathbf{F}_{ij}^{C}\boldsymbol{=}a_{ij}\sqrt{\omega\left( r_{ij} \right)}{\hat{\mathbf{r}}}_{ij}$$

where $a_{ij}$ is the repulsive parameter between the *i*th and *j*th beads. The $\mathbf{r}_{i}$ is the position of the *i*th bead, $\mathbf{r}_{ij}=\mathbf{r}_{i}-\mathbf{r}_{j}$, $r_{ij}=\left| \mathbf{r}_{ij} \right|$, and ${\hat{\mathbf{r}}}_{ij}=\mathbf{r}_{ij}/r_{ij}$. The weight function $\omega\left( r_{ij} \right)$ is set as

$$\omega\left( r_{ij} \right)=\left\{ \begin{matrix} \left( 1-{r_{ij}}/{r_{c}} \right)^{2} \\ 0 \end{matrix} \right., \begin{matrix} r_{ij}<r_{c} \\ r_{ij}>r_{c} \end{matrix}$$

and $r_{c}$ is the cut-off distance.

The dissipative force $\mathbf{F}_{ij}^{D}$, is a friction force that acts on the relative velocities of particles, defined as

$$\mathbf{F}_{ij}^{D}\boldsymbol{=-}\gamma\omega\left( r_{ij} \right)\left( {\hat{\mathbf{r}}}_{ij}\cdot\mathbf{v}_{ij} \right){\hat{\mathbf{r}}}_{ij}$$

where the $\gamma$ is the friction coefficient, $\mathbf{v}_{i}$ is the velocity of *i*th bead, and $\mathbf{v}_{ij}=\mathbf{v}_{i}-\mathbf{v}_{j}$.

The random force $\mathbf{F}_{ij}^{R}$ is given by

$$\mathbf{F}_{ij}^{R}\boldsymbol{=}\eta\sqrt{\omega\left( r_{ij} \right)}\theta_{ij}{\hat{\mathbf{r}}}_{ij}$$

where the $\theta_{ij}$ is the Gaussian white noise and the $\eta$ is the noise amplitude. The $\eta$ and $\gamma$ satisfy the fluctuation-dissipative theorem as

$$\eta^{2}=2\gamma k_{B}T$$

where the $k_{B}T$ is thermal energy.

In the DPD simulations, the reduced units are adopted for all physical quantities. The units of mass, length, and energy are $m$, $r_{c}$, and $k_{B}T$, respectively. The friction coefficient $\gamma$ and the noise amplitude$\eta$ are respectively set to be 4.5 and 3.0, and thus $k_{B}T=1.0$. The time unit $\tau$ can be obtained by

$$\tau=\sqrt{\frac{mr_{c}^{2}}{k_{B}T}}$$

and its real value can be estimated by matching the simulated lateral diffusion coefficient to the experiment measured value.

In the present work, the value of *n* is set as 2, 8, 11, and 17 to mimic the density variation of hydrogen bonds for the experimental systems. The backbone and each graft contain $N_{B}$ = 36 and $N_{G}$ = 2 beads, and the position of the *t*th graft points (denoted by *s_t_*) is located at

$$s_{t}=t\cdot\frac{N_{B}}{n+1}$$

In PAM-r-MVIC molecular chains, the interaction force between neighboring or bonded beads is considered as harmonic spring force,

$$\mathbf{F}_{ij}^{S}\boldsymbol{=}k_{b}\left( r_{ij}-r_{c} \right){\hat{\mathbf{r}}}_{ij}$$

where the spring constant is $k_{b}=100{k_{B}T}/{r_{c}}$, and the equilibrium length $r_{eq}=0.85 r_{c}$. The backbone (**B**) is solvophobic, while the grafts (**G**) are solvophilic. The repulsive parameters between different types of beads are listed in Table S1.

The hydrogen bonds between **G** grafts are described by the acceptor-hydrogen-donor (AHD) 3-body interaction potential based on the DREIDING force field

$$U^{\mathrm{AHD}}\left( r_{\mathrm{AD}} \right)=\left\{ \begin{matrix} 4k_{h}\left[ 5\left( \frac{1}{r_{\mathrm{AD}}} \right)^{12}-6\left( \frac{1}{r_{\mathrm{AD}}} \right)^{10} \right]{cos}^{4}\left( \theta_{\mathrm{AHD}} \right) \\ 0 \end{matrix} \right., \begin{matrix} for r_{\mathrm{AD}}<R_{c} and \theta_{\mathrm{AHD}}>\theta_{c} \\ else \end{matrix}$$

where the force constant $k_{h}$, cutoff distance $r_{c}$, and cutoff angle $\theta_{c}$ are set to be 20$k_{B}T$, 2.5$r_{c}$, and 150°, respectively. The hydrogen bonding interaction between a pair of **G** grafts exists only when the $r_{\mathrm{AD}}$ is within the cutoff distance $R_{c}=2.5r_{c}$ and the $\theta_{\mathrm{AHD}}$ is larger than the cutoff angle $\theta_{c}=150^{\circ}$. Otherwise, the hydrogen bonding interaction vanishes. Therefore, the hydrogen bonds described by the AHD potential are reversible due to their dynamical breaking and reforming. More importantly, directivity is introduced into the hydrogen bonds formed only in a limited range.

The initial cubic box of 30 × 30 × 30 ${r_{c}}^{3}$ with periodic boundary conditions was performed on the large atomic/molecular massively parallel simulator (LAMMPS). Total 81000 DPD beads with a polymer concentration of *ϕ* = 50% were randomly generated in the cubic box, where the *ϕ* is defined as the ratio of the polymer bead amount to the total bead amount. Firstly, a large number of DPD steps (6×10^5^) were carried out to capture equilibrated structures under the NVT ensemble with a time-step Δ*t*=0.001*τ* and the last 5×10^4^ steps with an interval of 5×10^3^ steps were collected for the subsequent extension tests. Then, the non-equilibrium extensions were implemented by a uniaxial deformation on the simulation box along *z*-direction with a constant strain rate and Δ*t* = 0.001*τ*. As the box is elongated in the *z*-direction, the box lengths in the *x* and *y* directions changed simultaneously to keep the density of the system constant. Therefore, the Poisson’s ratio is *μ* = 0.5, which is close to the rubbery materials and can be considered to have no volume change. The strain rate, the elongation in the *z*-direction (Δ*z*) divided by the box edge length *L_z_* (Δ*z*/*L_z_*), was set as 0.2/*τ*, where the strain rate is comparable with the segmental relaxation. The average tress *σ_z_* in the *z*-direction was calculated by the deviatoric part of the stress tensor

$$\sigma_{z}=\left( 1+\mu\right)\left( -\sigma_{zz}+P \right)={3\left( -\sigma_{zz}+P \right)}/2$$

where the $P=\sum_{\alpha} \left( {P_{\alpha\alpha}}/3 \right)$ is the static pressure. The diagonal component *P_αα_* of pressure tensor is the negative value of the average virial stress *σ_αα_* in the *α* direction calculated by the tensor version of the virial theorem

$$\sigma=\frac{1}{V}\left\langle\sum_{i} m_{i}\mathbf{v}_{i}\mathbf{v}_{i}\boldsymbol{+}\frac{1}{2}\sum_{i\neq j} \left( \mathbf{F}_{ij}\boldsymbol{\cdot}\mathbf{r}_{ij} \right) \right\rangle$$

where *V* is the volume of the simulation system and the angular bracket, $\left\langle\cdots\right\rangle$, represents the ensemble average. To eliminate the internal stress, the initial average stress *σ*_z,0_ should be subtracted from the average stress *σ_z_* to obtain the extension stress. Note that for a given parameter setting, eleven tensile tests on the above-collected structures were performed for an ensemble average of stress.

**S-4. Supplementary figures**


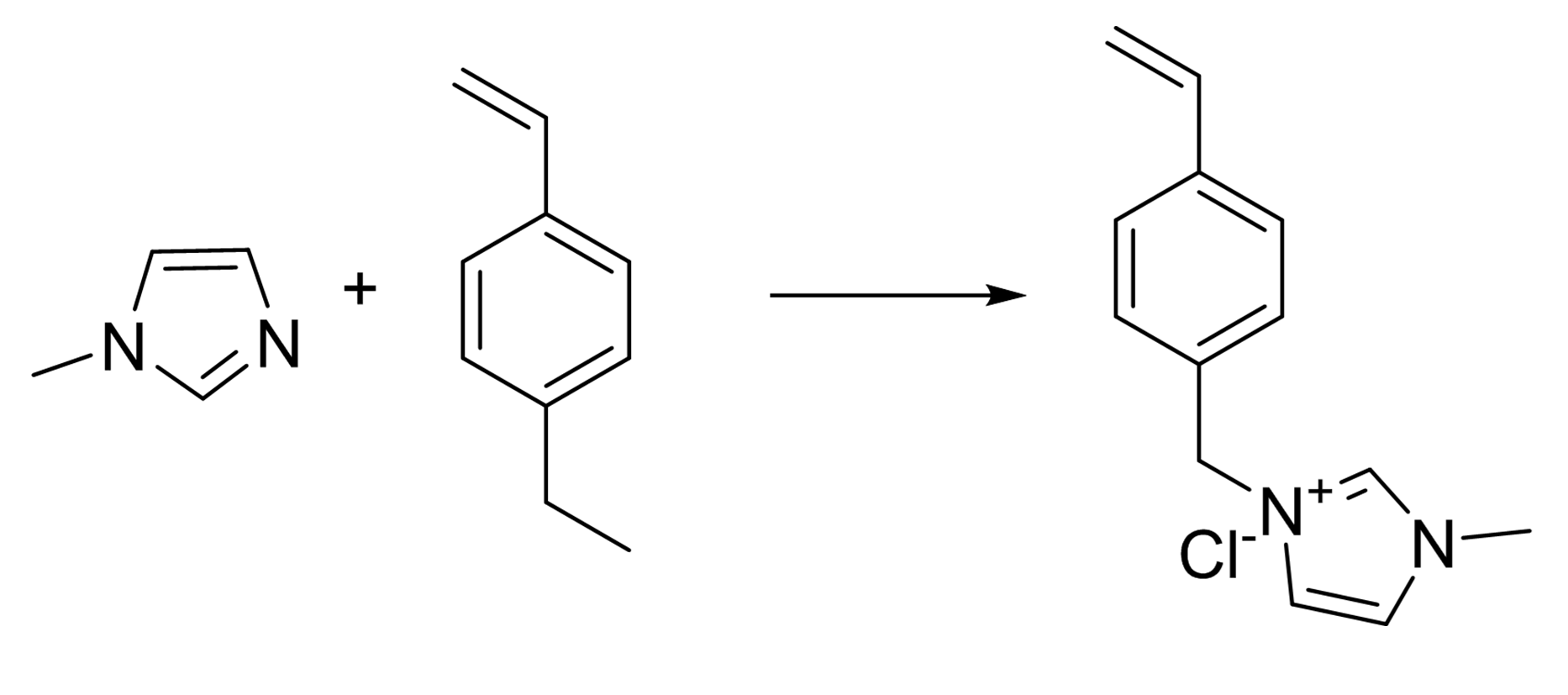


**Figure S1.** Synthesis route of the MVIC.


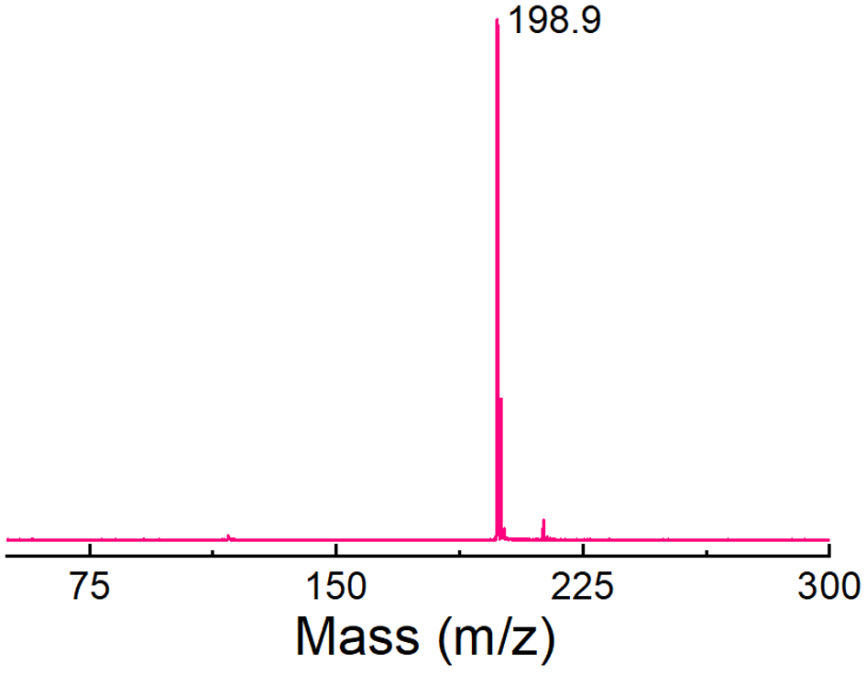


**Figure S2.** MALDI-TOF mass spectrum of the MVIC.


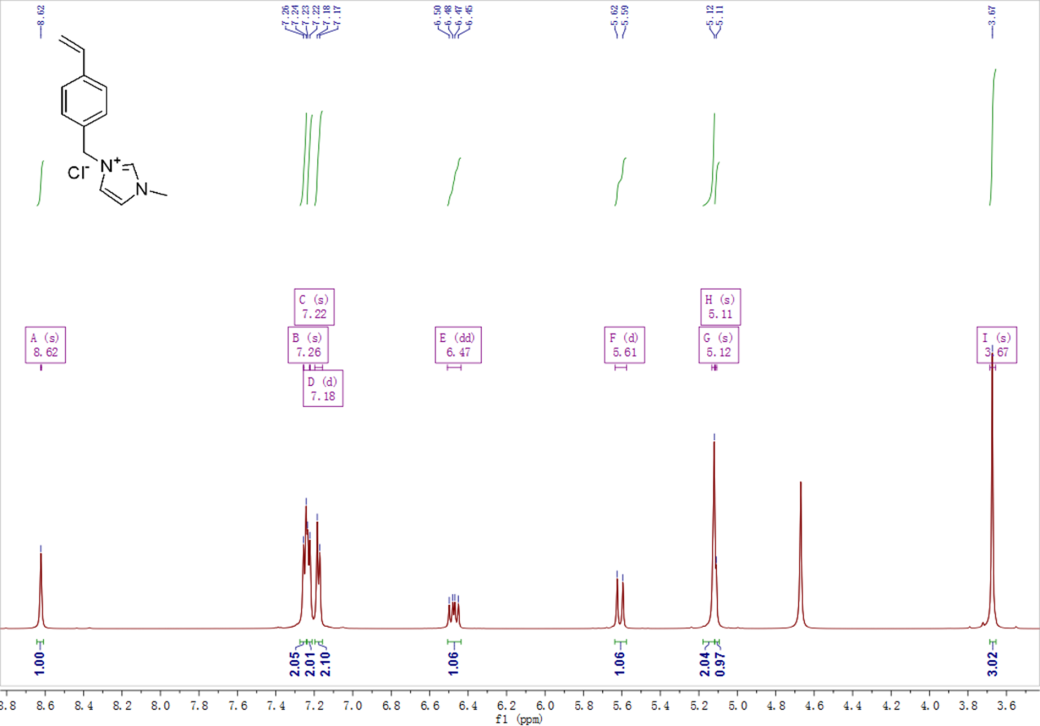


**Figure S3.** ^1^H NMR spectrum of MVIC in D_2_O.


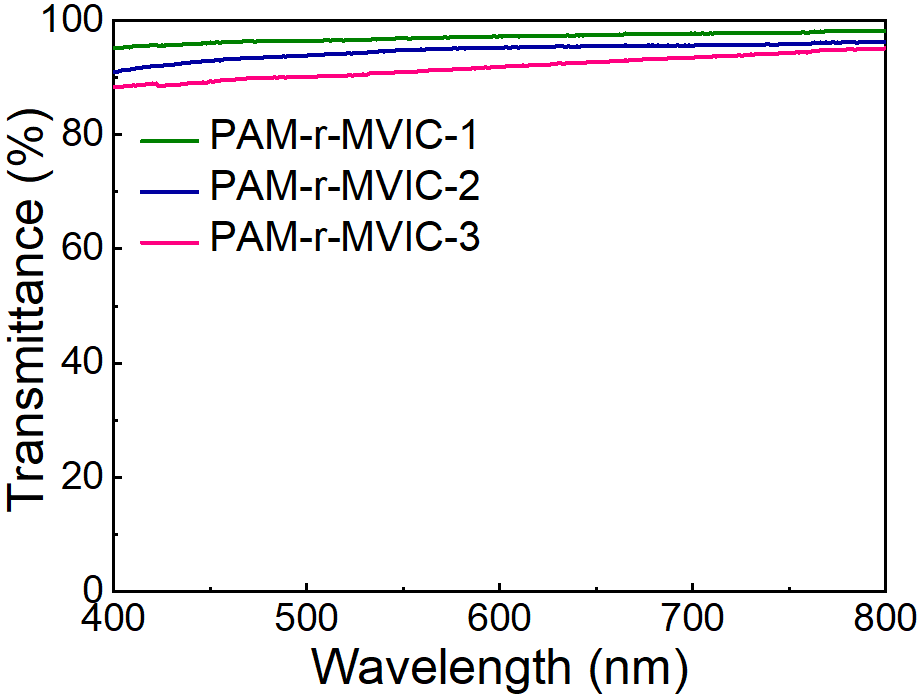


**Figure S4.** UV-vis spectra of the PAM-r-MVIC.


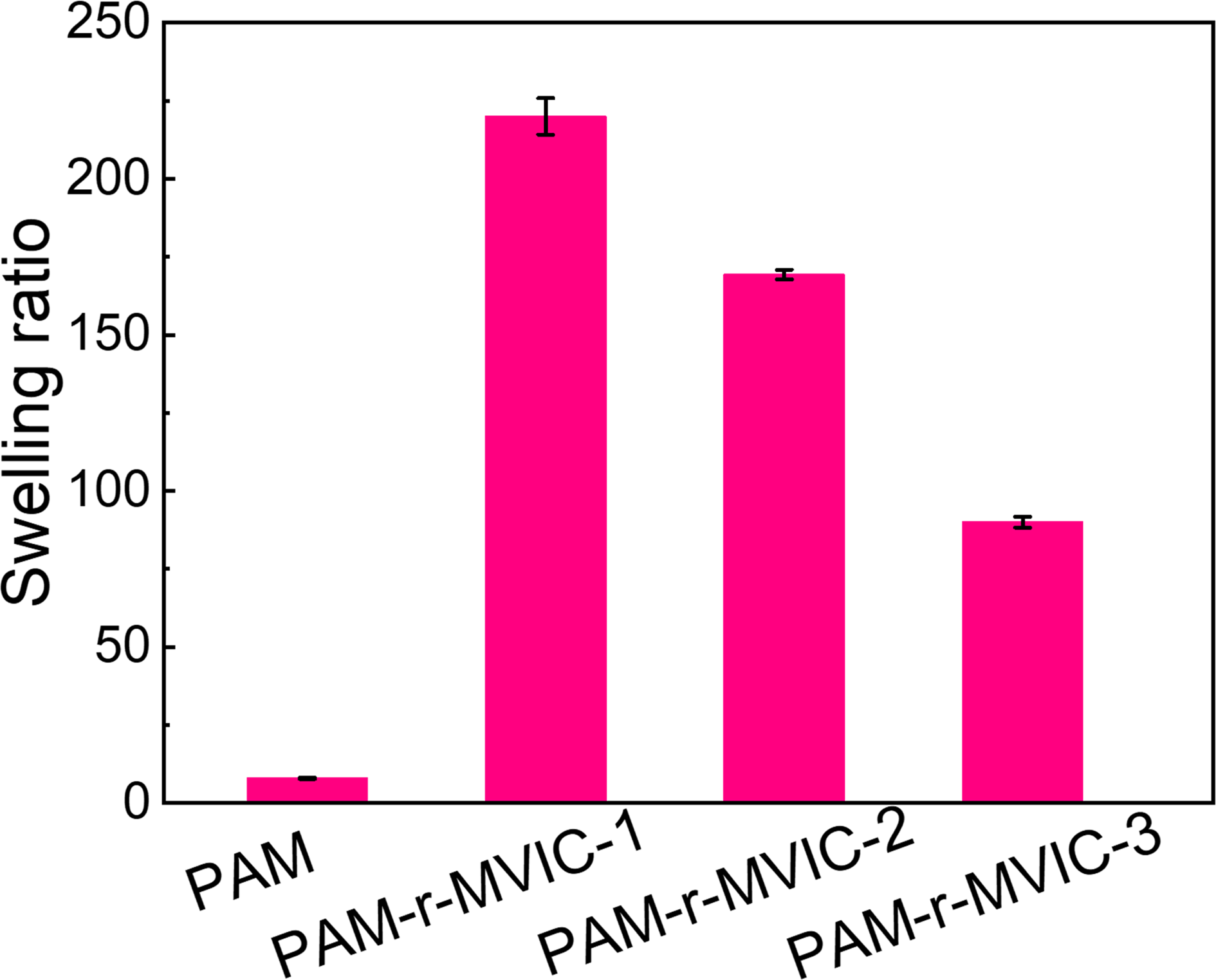


**Figure S5.** Equilibrium swelling ratios of the PAM-r-MVIC in water.


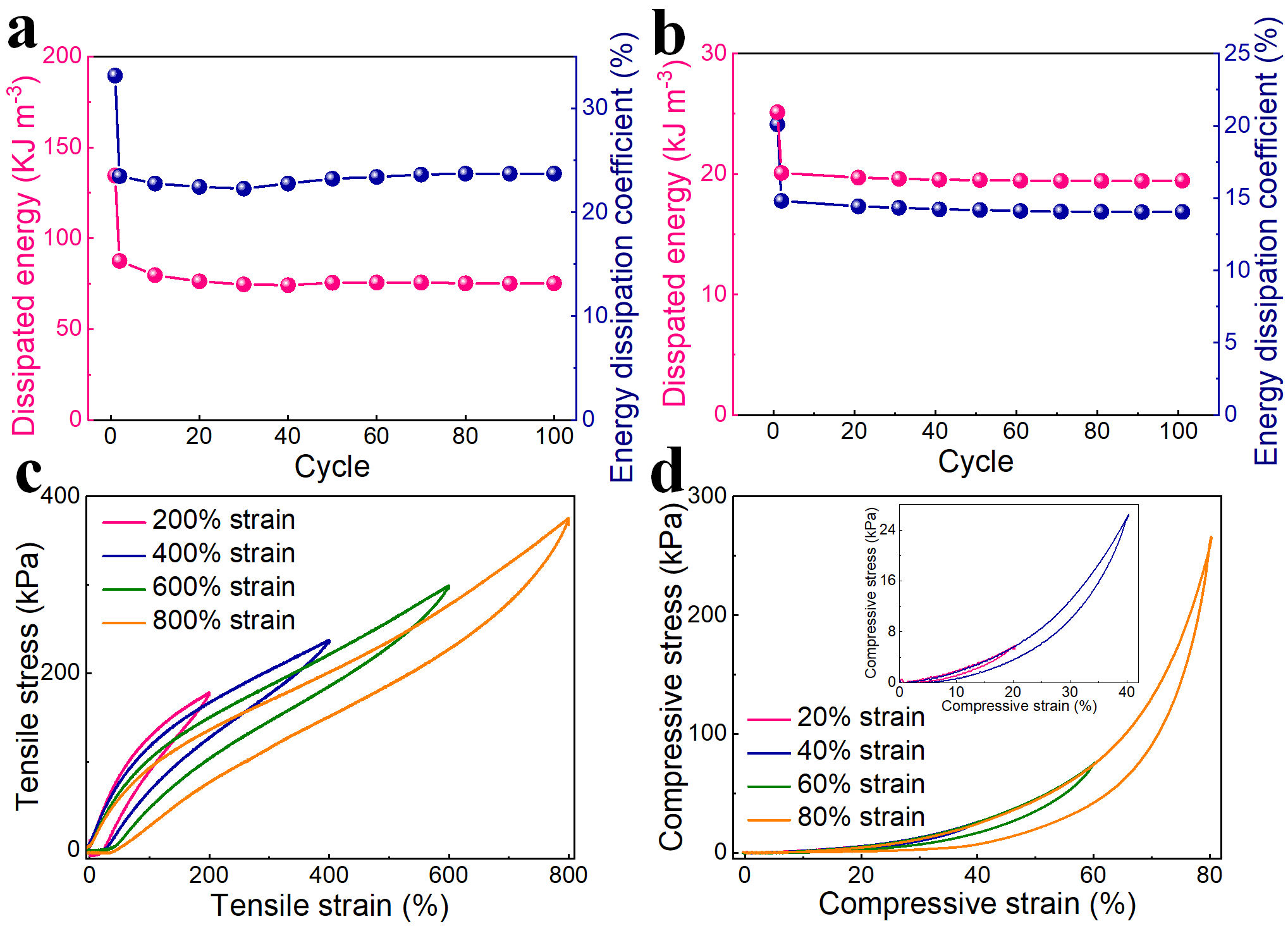


**Figure S6.** Successive loading-unloading tests of PAM-r-MVIC samples under tensile and compression. (a) Dissipated energy and energy dissipation coefficients of PAM-r-MVIC-2 under tensile loading-unloading cycles. (b) Dissipated energy and energy dissipation coefficients of PAM-r-MVIC-2 under compressive loading-unloading cycles. (c) Stress-strain curves of PAM-r-MVIC-2 at various tensile strains. (d) Stress-strain curves of PAM-r-MVIC-2 at various compressive strains. Small residual strain of PAM-r-MVIC-2 was observed when the successive tensile strain increased from 200% to 800% (Fig. S6c). The small hysteresis loops demonstrated a highly deformation-tolerant performance of PAM-r-MVIC-2, related to the presence of dense intermolecular hydrogen bonds. The same rule was also demonstrated in the successive loading-unloading compressive tests (Fig. S6d).


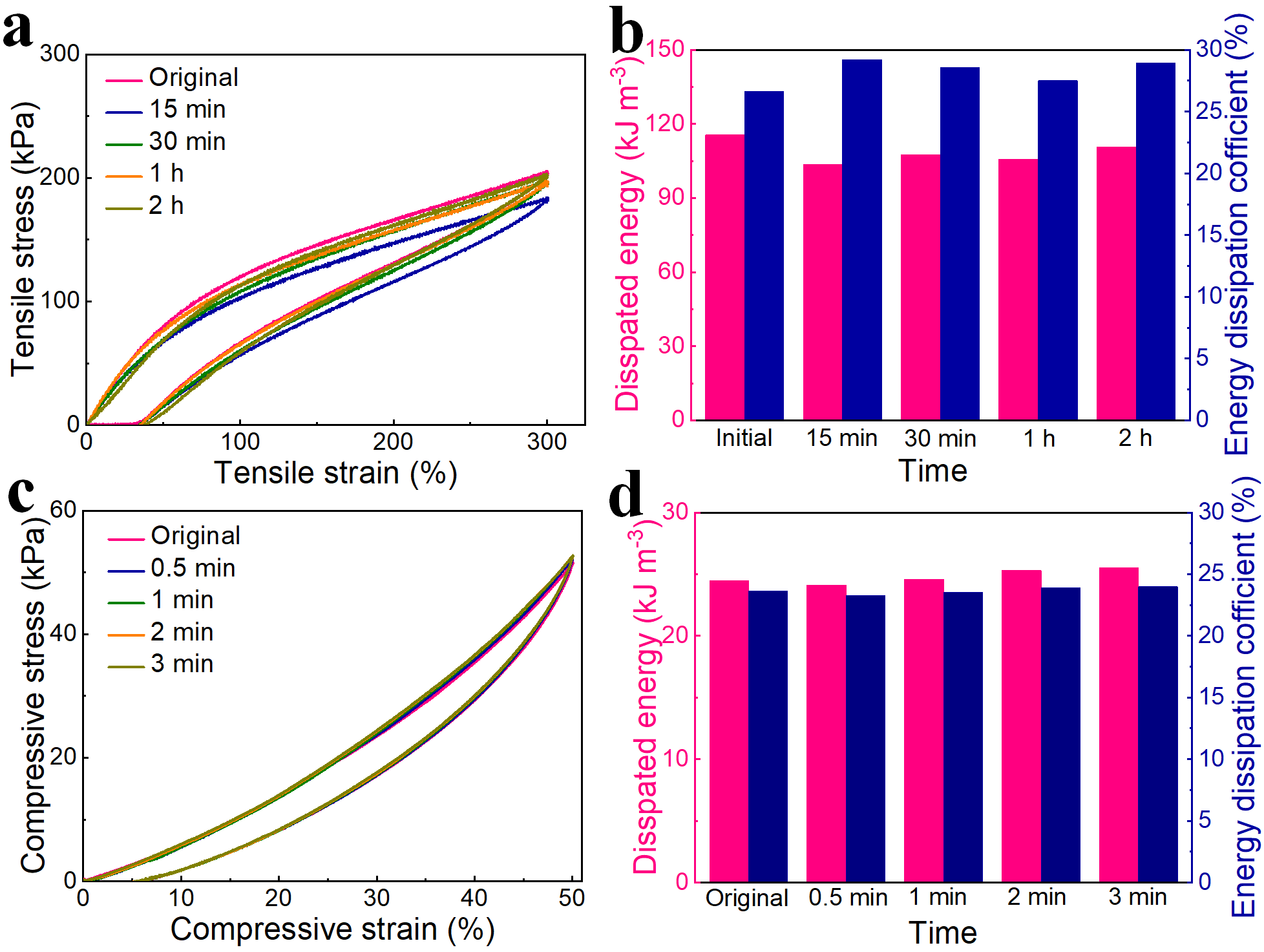


**Figure S7.** (a) Tensile recovery test of PAM-r-MVIC-2 with various resting time. (b) Tensile dissipated energy and energy dissipation coefficients during the tensile recovery test with various resting time. (c) Compressive recovery test of PAM-r-MVIC-2 with various resting time. (d) Tensile dissipated energy and energy dissipation coefficients during the compressive recovery test with various resting time.


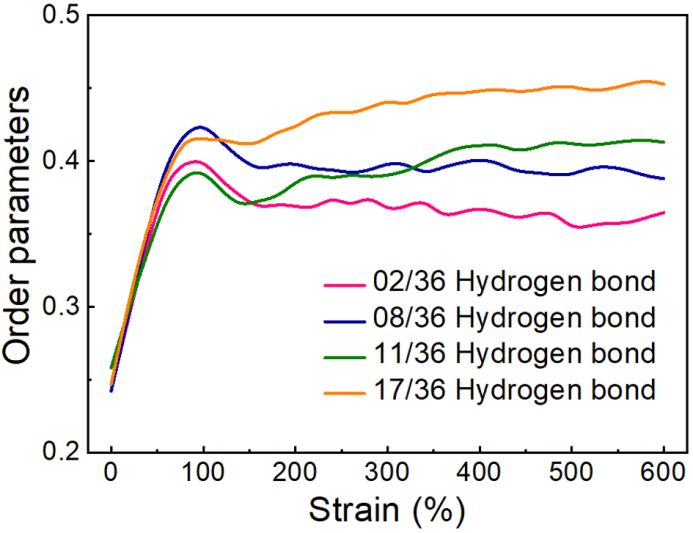


**Figure S8.** Order parameter of hydrogen bonds with various contents during stretching.


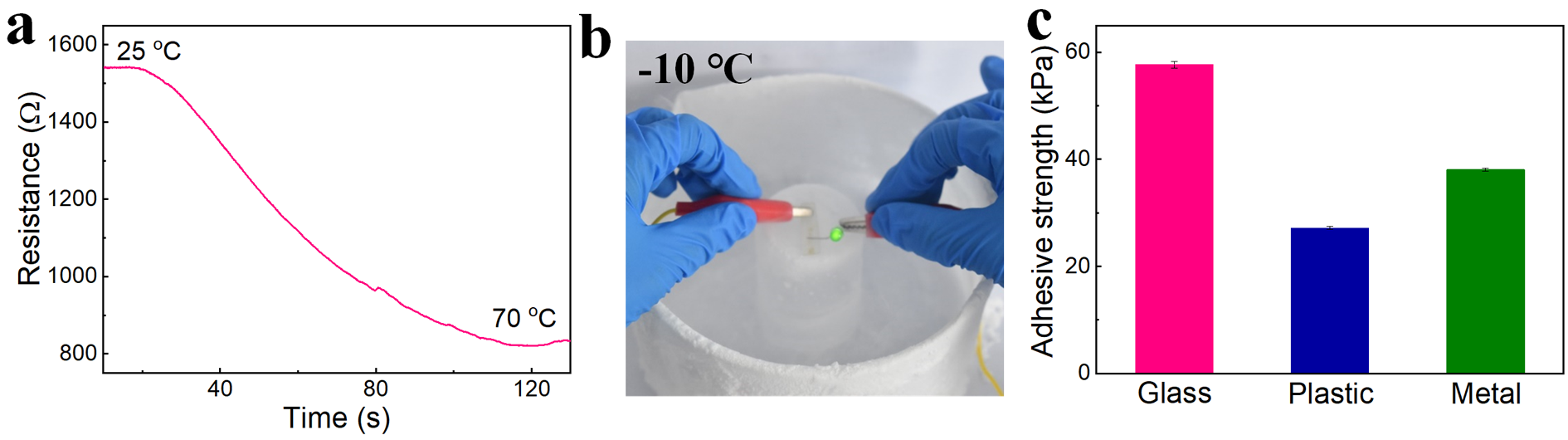


**Figure S9.** (a) Temperature-dependant resistance changes of PAM-r-MVIC-2. (b) Photograph showing the PAM-r-MVIC-2 lightening up an LED bulb at an extremely low temperature.

**Table S1.** Interaction parameters *a_ij_* (in DPD units) used in the simulations. The B, G, and S denote the backbone (**B**), graft (**G**), and solvent, respectively.

|  | B | G | S |
| --- | --- | --- | --- |
| B | 25 | 80 | 80 |
| G | 80 | 25 | 15 |
| S | 80 | 15 | 25 |

**Table S2.** Summary of mechanical properties and sensing performances of the PAM-r-MVIC and ionic conductive gels in literature.

| Samples | Tensile strength (kPa) / Strain (%) | Compressive strength (kPa) / Strain (%) | Sensing type | Working range | Gauge factor (Range) | Sensitivity (Range)  (kPa^-1^) | Ref. |
| --- | --- | --- | --- | --- | --- | --- | --- |
| PU-IL_2_ | 500/100 | - | Resistance | 0-300% | 1.23 (0-50%),  1.54 (50%-300%) | - | [6] |
| CG_2_-AAc_0.05_I_0.10_ gel | - | - | Resistance | 0-10 kPa | - | 0.33 (0-2 kPa),  0.01 (2-10 kPa) | [7] |
| Fe_3_O_4_@PAA/PAA ionogels | 12.5/800 | - | Resistance | 0-1400% | 3.82 (0-800%),  19.6 (800%-1400%) | - | [23] |
| MXene-PVA/PVP hydrogel | 150/500 | - | Resistance | 0-120 kPa | 1.81 (0-40 kPa),  3.29 (40-100 kPa) | - | [45] |
| HLP cross-linked hydrogels | 80/500 | 800/80 | Resistance | 0-150 kPa | - | 0.131 (0-13 kPa),  0.051 (13-50 kPa) | [47] |
| Binary-networked hydrogel | 25/500 | - | Resistance | 0-100% | 0.66 (0-20%),  0.71(20-60) | - | [50] |
| **PAM-r-MVIC-2** | **370/800** | **264/80** | **Capacitance**  **/Resistance** | **C: 0-10 kPa;**  **R: 0-120 kPa** | **-** | **C: 0.06 (0-3 kPa),**  **0.04 (3-10 kPa);**  **R: 0.01 (0-60 kPa),**  **0.15 (60-120 kPa)** | **This work** |
